# Prevalence of non-obstructive dysphagia in patients with heartburn and regurgitation

**DOI:** 10.6061/clinics/2020/e1556

**Published:** 2020-01-20

**Authors:** Andrea Oliveira Batista, Weslania Viviane Nascimento, Rachel Aguiar Cassiani, Ana Cristina Viana Silva, Leda Maria Tavares Alves, Dauana Cássia Alves, Roberto Oliveira Dantas

**Affiliations:** Faculdade de Medicina de Ribeirao Preto, Universidade de Sao Paulo, Ribeirao Preto SP, BR

**Keywords:** Dysphagia, Gastroesophageal Reflux, Deglutition, Deglutition Disorders, Heartburn, Esophagitis

## Abstract

**OBJECTIVE:**

Heartburn and regurgitation are the most common gastroesophageal reflux symptoms, and dysphagia could be a possible symptom. This investigation aimed to evaluate the prevalence of non-obstructive dysphagia in patients with heartburn and regurgitation.

**METHODS:**

A total of 147 patients (age, 20-70 years; women, 72%) complaining of heartburn and regurgitation, without esophageal stricture, previous esophageal surgery, or other diseases, were evaluated. Twenty-seven patients had esophagitis. The Eating Assessment Tool (EAT-10) was employed to screen for dysphagia; EAT-10 is composed of 10 items, and the patients rate each item from 0 to 4 (0, no problems; 4, most severe symptom). Results of the 147 patients were compared with those of 417 healthy volunteers (women, 62%; control group) aged 20-68 years.

**RESULTS:**

In the control group, only two (0.5%) had an EAT-10 score ≥5, which was chosen as the threshold to define dysphagia. EAT-10 scores ≥5 were found in 71 (48.3%) patients and in 55% of the patients with esophagitis and 47% of the patients without esophagitis. This finding indicates a relatively higher prevalence of perceived dysphagia in patients with heartburn and regurgitation and in patients with esophagitis. We also found a positive correlation between EAT-10 scores and the severity of gastroesophageal reflux symptoms based on the Velanovich scale.

**CONCLUSION:**

In patients with heartburn and regurgitation symptoms, the prevalence of dysphagia was at least 48%, and has a positive correlation with the overall symptoms of gastroesophageal reflux.

## INTRODUCTION

Gastroesophageal reflux disease (GERD) is prevalent worldwide (1,2), and its incidence among young individuals has been increasing (1). Although the most frequent symptoms are heartburn and regurgitation, other symptoms, such as dysphagia, odynophagia, globus sensation, chest pain, belching, chronic cough, laryngitis, hoarseness, and asthma, may be present (3,4).

Dysphagia means difficulty in swallowing that may occur in the oral, pharyngeal, or esophageal phases of swallowing (5). Non-obstructive GERD is the most common identifiable cause of esophageal dysphagia (6,7) and the major cause in younger individuals. A previous study reported that non-obstructive GERD was observed in approximately 24% of the patients who sought treatment for dysphagia (6). Intermittent dysphagia is independently associated with GERD (8), and 31.6% and 18% of patients with GERD have frequent and infrequent dysphagia, respectively (7). Moreover, 37-46.8% of patients with esophagitis reported dysphagia in previous studies (9,10).

Symptom descriptions based on self-reports with dichotomized answers, i.e., yes or no, are not always precise (11) and may be influenced by culture, beliefs, and ethnicity (12). Thus, we used the validated instrument Eating Assessment Tool (EAT-10), which evaluates dysphagia based on an individual’s perception, to determine the prevalence of dysphagia in patients with heartburn and regurgitation in Brazil.

EAT-10 is a quick, easy-to-complete, non-invasive, inexpensive, and self-administered screening tool, which is based on patient’s self-perception, to detect possible swallowing impairment before performing a more specific examination (13). This instrument has good internal consistency and test-retest reproducibility and validity (13,14), with a sensitivity of 0.85 and specificity of 0.82 to detect dysphagia (15); moreover, it has the ability to predict aspiration (16-20). EAT-10 is recommended as the first-line screening tool for at-risk patients (15), has been validated in different languages, and is currently used for dysphagia evaluation in different populations (14,21-30). In addition, while this tool could be used for both oropharyngeal and esophageal dysphagia, it is most frequently used to detect the former (13). Each of the 10 questions can be rated from 0 to 4 (0, no problem; 4, severe swallowing problem). Previous investigations found that EAT-10 scores ≥3 is indicative of dysphagia (13,14,23,26), and this cut-off value has a sensitivity of 0.76 and specificity of 0.75 (31).

This study hypothesized that dysphagia is highly prevalent in patients with heartburn and regurgitation and is associated with the overall gastroesophageal reflux symptoms.

## MATERIALS AND METHODS

In this study, EAT-10 was administered to 147 patients (106 [72%] women, 41 [28%] men) aged 20-68 years (mean 43.2 [13.2] years) who had heartburn and regurgitation (Table 1). The patients completed the instrument just before endoscopic examination in a public tertiary hospital. They also responded to the questionnaire on reflux symptoms proposed by Velanovich et al. (32), which was translated to Portuguese (33). Each of the 10 items in the questionnaire could be rated from a 0 to 5 (0, no symptom; 5, incapacitating symptom), with a possible total rating ranging from 0 to 50. None of the patients had esophageal stricture, history of previous surgery of the upper digestive tract, or other diseases, and they did not receive proton pump inhibitors regularly.

Endoscopic examination revealed esophagitis in 25 patients (11 Los Angeles classification (LA) grade A (34), 11 LA grade B, two LA grade C, and one LA grade D), and two patients had Barrett’s esophagus. No endoscopic abnormality was found in the remaining 120 patients; moreover, none of the patients had pharyngeal or esophageal strictures, cancer, or eosinophilic esophagitis based on endoscopic findings. Twenty-four-hour intraesophageal pH monitoring was performed in 38 patients, with a pH probe placed 5 cm above the lower esophageal sphincter after manometry. Excessive reflux was considered when the acid exposure time (intraesophageal pH <4) was greater than 6% of the monitoring time (35).

EAT-10 was also administered to 417 healthy individuals (control group). These individuals had no disease, symptoms, or previous surgery of the upper digestive tract and were not treated for any disease. This group was composed of 257 (62%) women and 160 (38%) men aged 20 to 70 years (mean 37.9 [14.3] years).

This study was approved by the Human Research Committee of the university hospital (IRB numbers 9635/2013 and 12220/2016). Written informed consent was obtained from each participant, and anonymity of all participants was guaranteed. The EAT-10 questionnaire was translated from the original version to Brazilian Portuguese and validated (21) (Table 2).

Statistical analysis was performed by non-parametric tests, the Mann-Whitney test, Kruskal-Wallis test with pos-test of Dunn, and Spearman test of correlation. The program used was SAS 9.2 (SAS Inst., Cary NC, 2011). The results are shown in mean, standard deviation, median, correlation coefficient (rho), and percentage. A *p* value ≤0.05 was considered significant.

## RESULTS

**Figure 1:**
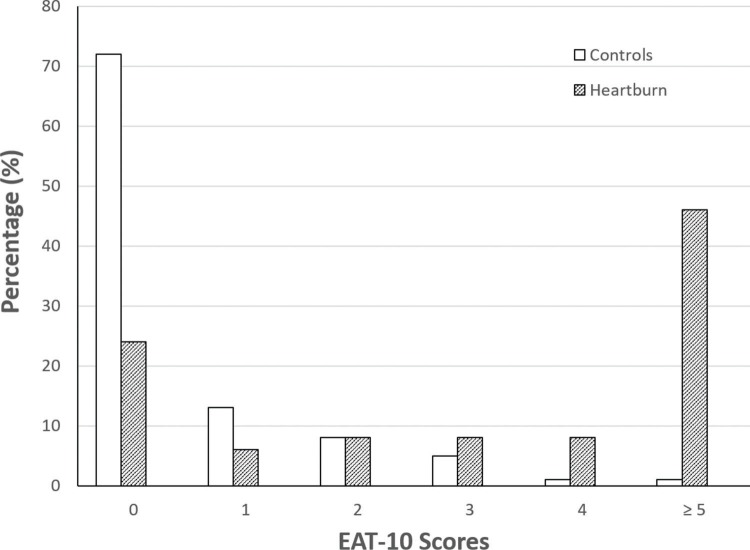
Eating Assessment Tool (EAT -10) scores.

Figure 1 shows the EAT-10 scores in the control and study groups. The total score ranged from 0 to 8 (mean 0.59, median 0) in the control group and from 0 to 37 (mean 9.2, median 4) in the study group (Table 1). If we consider EAT-10 scores ≥3 as the threshold to define dysphagia, 29 controls (6.9%) and 95 patients (64.6%) had dysphagia. Almost all controls (99.3%) had EAT-10 scores ≤4, which we used as the threshold to define dysphagia in our study. Using this threshold, the number of patients with dysphagia was 71(48.3%). Mean EAT-10 score in patients with dysphagia (EAT-10 ≥5) was 17.5, and the median was 18 (range 5-37).

Moreover, mean EAT-10 score was 10.0 (9.9) in patients with esophagitis and 9.1(10.2) in patients without esophagitis (*p*>0.05). EAT-10 score ≥5 was noted in 56% of the patients with esophagitis and 47% of the patients without esophagitis (*p*>0.05). A positive correlation between EAT-10 scores and Velanovich scores was found (*p*<0.01; Table 3). Mean Velanovich score was 30.3 (10.2), with a median of 31.

In addition, 20% of the patients who performed the manometric examination had a diagnosis of ineffective esophageal motility, with no association with EAT-10 scores (*p*>0.05), which suggested that a higher EAT-10 score may be observed in patients with or without ineffective esophageal motility.

## DISCUSSION

Based on the threshold of EAT-10 score ≥3, 64.6% of the patients with heartburn and regurgitation had dysphagia, and using the threshold EAT-10 score of ≥5, we found that 48.3% of the patients have dysphagia. These findings indicated a high frequency of dysphagia among the patients evaluated in Brazil.

In the patients included in this study, several conditions may have been associated with heartburn and regurgitation, including erosive GERD, non-erosive GERD, reflux hypersensitivity, and functional heartburn (36). Although heartburn and regurgitation are the most frequent symptoms of GERD, the sensitivity and specificity of these symptoms for the identification of GERD are insufficient (sensitivity, 65%; specificity, 75%) (2,4).

The presence of dysphagia in patients with heartburn and regurgitation may be explained by the following:

- Upper esophageal sphincter (UES) dysfunction.
  Gastroesophageal reflux could influence UES function. Patients with GERD have longer UES opening during deglutition, which means a longer time for the bolus to pass through the sphincter (37,38). Other UES changes have been described, such as short and hypotonic sphincter (39) and increased UES pressure associated with transient lower esophageal sphincter relaxation (40). Reflux events cause an intraesophageal pressure increase, which evokes UES contractile response (41). Chronic acid exposure in the esophageal body could cause hypertonicity of the UES and thus difficulty in opening (42). The UES opening diameter during swallowing was smaller in patients with than in those without hiatal hernia (43). Slower passage of the bolus through the UES has been associated with dysphagia in patients with esophagitis (38), and a slower bolus transit through the pharynx has also been reported in the disease (37,38). A recent investigation found that patients with reflux-associated dysphagia have delayed airway closure relative to the arrival of the bolus at the UES, suggesting a delay in airway protection when the bolus is already in the pharynx (44).
- Hypersensitivity
  Some patients with heartburn may have abnormal esophageal sensitivity to acid (reflux hypersensitivity), which is characteristic of a functional esophageal disorder (45). These patients have increased chemo- and mechano receptor sensitivity to acid perfusion and balloon distention (45). They manifest GERD symptoms during reflux even in the absence of abnormal acid exposure or esophagitis (46,47). In functional heartburn, the symptoms are not associated with gastroesophageal reflux. Nevertheless, functional esophageal disorders are associated with peripheral sensitization, central sensitization, and viscera-visceral hyperalgesia and cause a significant reduction in the quality of life (46,48). Such hypersensitivity related to esophageal innervations (46) may increase patients’ perception of esophageal bolus transit during swallows, and stress, anxiety, and hypervigilance may have a role in the development of esophageal hypersensitivity (49). In addition, calcitonin gene-related positive nerves, which is a marker of nociceptive sensory innervation, are more superficial in the proximal and distal esophagus of patients with non-erosive reflux disease, which may contribute to symptoms during swallows (50).
- Esophageal dismotility
  GERD may be the cause or the consequence of esophageal dismotility (51,52). The frequency and intensity of esophageal motility abnormalities increase with the severity of reflux disease (52). Transient lower esophageal sphincter relaxation followed by reflux, hypotensive lower esophageal sphincter, ineffective esophageal peristalsis, and bolus transit abnormalities are strongly implicated in GERD (53). High-resolution esophageal manometry during solid swallows demonstrated motility abnormality in patients with a non-erosive disease, including ineffective swallows, large breaks, and decreased distal contractile integral, leading to a delay in acid clearance (53). Excessive esophageal acid exposure with reduced esophageal peristaltic contractions may be seen in a high proportion of patients with dysphagia, even in those without heartburn and regurgitation (49). These esophageal motility changes may be the cause of non-obstructive dysphagia (54).

Results of the EAT-10 were associated with the severity of gastroesophageal reflux symptoms, indicating that the intensity of overall symptoms is related to dysphagia severity. Depressive disorders are frequently comorbid with GERD (55) and may also be related to the intensity of the symptoms, including the perception of dysphagia.

Previous investigations found that the odds ratio for patients with heartburn to have frequent dysphagia is 5.9 and that for patients with acid regurgitation is 10.6 (7). Among patients with dysphagia, 58% had heartburn, 67% had regurgitation, and 72% had GERD, and an association between intermittent dysphagia and GERD was observed (odds ratio, 2.96) (8). Functional dysphagia is not the most probable diagnosis because in the Rome IV criteria, GERD is an exclusion criterion for functional dysphagia, which is the least prevalent among functional esophageal disorders (48). Although the definition of dysphagia and the methods of evaluation may differ and thus yield different results, the number of patients with symptomatic dysphagia is usually high, suggesting that, after endoscopy, esophageal manometry with provocation testing is an essential examination to determine the etiology of dysphagia.

This investigation has some limitations. The number of patients with an erosive disease was small; however, it still reflects the real-world situation, as only 30% of patients with heartburn have an erosive disease (35). The 24-h pH monitoring was not performed in all subjects because of technical and cost limitations. The patients’ treatment for GERD with proton pump inhibitors was not regular; they were instructed to stop receiving the medications 1 week before endoscopy and pH monitoring. Adequate treatment for GERD decreases the frequency and severity of dysphagia (9) and improves esophageal motility (49).

Therefore, heartburn and regurgitation are associated with dysphagia, with somatization as a risk factor for non-obstructive dysphagia (57). The complaint of dysphagia in patients with no previous dysphagia may be a manifestation of a complication, such as a benign or malignant esophageal stricture. An anatomic cause of dysphagia, such as diverticula or hiatal hernias, may not be seen on endoscopy, however, it is unlikely to occur in these patients because they received medical attention and evaluations before the indication of endoscopy.

## CONCLUSION

Using an EAT-10 score ≥5 as the threshold to define dysphagia, we found that 48% of patients with heartburn and regurgitation have dysphagia. This symptom has a positive correlation with the overall symptoms of gastroesophageal reflux, which suggested that the presence and intensity of dysphagia are related to the symptoms due to GERD.

## AUTHOR CONTRIBUTIONS

Batista AO participated in the study planning, investigation, data collection, discussion of results, contributed to the manuscript preparation and the decision to submit the manuscript for publication. Nascimento WV, Cassiani RA, Silva ACV, Alves LMT and Alves DC participated in the data collection, discussion of results, contributed to the manuscript preparation and the decision to submit the manuscript for publication. Dantas RO participated in the study planning, discussion of results, manuscript preparation and decision to submit the manuscript for publication.

## Figures and Tables

**Table 1:** Characteristics of the groups evaluated by the Eating Assessment Tool (EAT-10).

	Control group	Study group
Number (n)	417	147
Women (%)	61.6	72.1
Age (years)	37.9 (14.3)	43.2 (13.2)
Height (m)	1.67 (0.09)	1.63 (0.09)
Weight (kg)	80.3 (26.7)	76.5 (15.3)
BMI (kg/m^2^)	28.8 (9.2)	28.8 (5.6)
Velanovich score	_	30.3 (10.2)
EAT-10 score	0.59 (1.2)	9.2 (10.1)*

BMI, body mass index

Data are expressed as mean (SD).

* *p*<0.01 *vs*. controls

**Table 2:** Eating Assessment Tool (EAT-10) in Brazilian Portuguese.

O quanto as seguintes situações são problemáticas para o senhor (a) (To what extent are the following scenarios problematic for you)	0 = Sem problema (No problem) 4 = Grave problema (Severe problem)				
1. Meu problema para engolir me faz perder peso (My swallowing problem has caused me to lose weight)	0	1	2	3	4
2. Meu problema para engolir não me deixa comer fora de casa (My swallowing problem interferes with my ability to go out for meals)	0	1	2	3	4
3. Preciso fazer força para beber liquido (Swallowing liquids takes extra effort)	0	1	2	3	4
4. Preciso fazer força para engolir comida (sólidos) (Swallowing solids takes extra effort)	0	1	2	3	4
5. Preciso fazer força para engolir remédios(Swallowing pills takes extra effort)	0	1	2	3	4
6. Dói para engolir (Swallowing is painful)	0	1	2	3	4
7. Meu problema para engolir me tira o prazer de comer (The pleasure of eating is affected by my swallowing)	0	1	2	3	4
8. Fico com comida presa/entalada na garganta (When I swallow, food sticks in my throat)	0	1	2	3	4
9. Eu tusso quando como (I cough when I eat)	0	1	2	3	4
10. Engolir me deixa estressado (Swallowing is stressful)	0	1	2	3	4
Total EAT-10:					

**Table 3:** Correlation of age, height, body mass index, and Velanovich score with EAT-10 scores (Spearman’s correlation coefficient [rho]).

	Control group (n=417)				Study group (n=147)			
	rho	95% CI		*p*	rho	95% CI		*p*
Age	-0.23	-0.32	-0.13	<0.01	0.05	-0.11	0.21	0.52
Height	-0.05	-0.14	0.05	0.34	-0.20	-0.35	-0.04	0.01
BMI	-0.13	-0.22	-0.04	<0.01	-0.09	-0.25	0.07	0.26
Velanovich	-	-	-	-	0.59	0.45	0.69	<0.01

BMI, body mass index; CI, confidence interval.
